# Gestational Outcomes in Patients with Severe Maternal Morbidity Caused by Hypertensive Syndromes

**DOI:** 10.1055/s-0040-1701464

**Published:** 2020-02

**Authors:** Daisy de Lucena Feitosa Lins Pinheiro, Francisco Edson de Lucena Feitosa, Edward Araujo Júnior, Francisco Herlânio Costa Carvalho

**Affiliations:** 1 Department of Obstetrics and Gynecology, Faculdade de Medicina, Universidade Federal do Ceará, Fortaleza, CE, Brazil; 2 Department of Obstetrics, Escola Paulista de Medicina, Universidade Federal de São Paulo, São Paulo, SP, Brazil; 3 Medical Course, Universidade Municipal de São Caetano do Sul, São Paulo, SP, Brazil

**Keywords:** pregnancy complications, maternal morbidity, high-risk pregnancy, hypertensive disorders, complicações na gestação, morbidade materna, gestação de alto risco, distúrbios hipertensivos

## Abstract

**Purpose** To evaluate the impact of the presence of criteria for severe maternal morbidity and maternal near miss associated with hypertensive disorders on maternal and perinatal outcomes in a maternity school.

**Methods** The present is a sub-analysis of a larger study involving 27 centers in Brazil that estimated the prevalence of serious maternal morbidity and near miss. It is an analytical and cross-sectional study with a quantitative approach, involving 928 women who were cared for at Maternidade Escola Assis Chateaubriand (MEAC, in Portuguese), Universidade Federal do Ceará (UFC, in Portuguese), from July 2009 to June 2010. The women were diagnosed with near miss according to the World Health Organization (WHO) criteria. The sample was divided into 2 groups: patients with (n = 827) and without hypertension (n = 101). The results were considered statistically significant when *p* < 0.05. The Pearson chi-squared and Fisher Exact tests were used for the categorical variables, and the Mann–Whitney U test was used for the continuous variables.

**Results** In total, 51 participants with maternal near miss criteria were identified, and 36 of them had hypertensive disorders. Of these, 5 died and were obviously excluded from the near miss final group. In contrast, we observed 867 cases with non-near miss maternal morbidity criteria. During this period, there were 4,617 live births (LBs) in the institution that was studied.

**Conclusion** In the severe morbidity/maternal near miss population, the presence of hypertensive complications was prevalent, constituting a risk factor for both the mother and the fetus.

## Resumo

**Objetivo** Avaliar o impacto da presença de critérios de morbidade materna grave e potencial evento adverso materno associados a distúrbios hipertensivos nos desfechos maternos e perinatais em uma maternidade escola.

**Métodos** Trata-se de uma subanálise de um estudo maior, envolvendo 27 centros, que estimou a prevalência de morbidade materna grave e potencial evento adverso no Brasil. Realizou-se um estudo analítico e transversal, com abordagem quantitativa, envolvendo 928 mulheres atendidas na Maternidade Escola Assis Chateaubriand (MEAC), na Universidade Federal do Ceará (UFC), no período de julho de 2009 a junho de 2010, diagnosticadas com potencial evento adverso de acordo com os critérios da Organização Mundial da Saúde (OMS). A amostra foi dividida em dois grupos: pacientes com (n = 827) e sem hipertensão (n = 101). Os resultados foram considerados estatisticamente significativos quando *p* < 0,05. O teste do Qui-quadrado de Pearson e o teste exato de Fisher foram usados para as variáveis categóricas, e o teste U de Mann-Whitney, para as variáveis contínuas.

**Resultados** Foram identificados 51 participantes com critérios de potencial evento adverso materno, sendo 36 mulheres com distúrbios hipertensivos. Destas, 5 morreram e foram obviamente excluídas do grupo final do potencial evento adverso. Foram observados 867 casos com critérios de morbidade materna que não caracterizavam potencial evento adverso. Nesse período, houve 4.617 nascidos vivos (NVs) na instituição estudada.

**Conclusão** Na população com morbidade grave/potencial evento adversomaterno, a presença de complicações hipertensivas é prevalente, constituindo fator de risco para o binômio materno-fetal.

## Introduction

Arterial hypertension is a severe public health problem, and it is a frequent cause of gestational complications worldwide.^1^ ^2^ ^3^ Hypertensive disorders have the potential to increase other clinical complications 3- to 25-fold, which may lead to death.^4^ ^5^

Hypertensive disorders are responsible for ∼ 50 thousand cases of global maternal deaths per year, and they are the main cause of maternal death in Latin America and the Caribbean. Estimates indicate that this problem represents a quarter of the total number of these deaths.^6^ ^7^ In Brazil, it is the primary cause of direct maternal death, with a prevalence of 37% of these cases; additionally, the ratio is higher in the Northern and Northeastern regions of Brazil compared with the Southeastern, Southern and Midwestern regions.^7^ ^8^ ^9^

Studying maternal death is a step to improve the obstetrical care process, as it can signal potential problems in the health system and enable the development of strategies to improve the quality of maternal assistance.^10^ However, an important piece of data to increase the information related to maternal health is the identification and analysis of cases involving women who survived severe complications during pregnancy, parturition and the puerperium – which are called maternal near miss –, because they allow us to understand the aspects that the survivors have in common with those who died from these complications, facilitating the gathering of information. The patients themselves are sources of information about what truly happened during their medical care, reporting potential difficulties with referrals and delayed care.^11^ ^12^

Near misses occur more often than maternal death. The identification and knowledge of these cases enables the development of strategies to reduce the number of maternal deaths for reasons that could be avoided.^13^

The present study aimed to evaluate the impact of the presence of criteria for severe maternal morbidity and maternal near miss associated with hypertensive disorders on maternal and perinatal outcomes in a maternity school that is a reference in tertiary care.

## Methods

The present is an analytical and cross-sectional study with a quantitative approach, involving 928 women who were cared for at Maternidade Escola Assis Chateaubriand (MEAC, in Portuguese), Universidade Federal do Ceará (UFC, in Portuguese), from July 2009 to June 2010; the patients were diagnosed with near miss according to the World Health Organization (WHO)^14^ criteria and/or the non-near miss maternal morbidity criteria of the Brazilian Ministry of Health. Severe morbidity cases due to abortion were excluded, cases in which the parturition did not occur in the institution, as well as cases in which the medical records were unavailable for consulting. This is a sub-analysis of a larger study involving 27 centers that estimated the prevalence of severe maternal morbidity and near miss in Brazil.^15^

The variables that were studied include: age, schooling, race/ethnicity, marital status, number of pregnancies, parity, previous abortion, previous cesarean section, process of parturition, preexistent clinical conditions, perinatal outcome (birth weight, Apgar score and death) and maternal outcome (severe maternal morbidity, including near miss and maternal death).

The sample was divided in two groups: patients with (n = 827) and without hypertension (n = 101). Out of the 827 participants who had a diagnosis of hypertensive syndrome, 49 had chronic hypertension, 20 had preeclampsia without signs of severity, 616 had preeclampsia with signs of severity, 60 had eclampsia, and 82 had hemolysis, elevated liver enzymes, and a low platelet count (HELLP) syndrome. Chronic hypertension was considered when the hypertension diagnosis (≥ 140 × 90 mm Hg) occurred before the 20th gestational week in the absence of proteinuria. Preeclampsia was considered when hypertension was identified after the 20th gestational week in the presence of proteinuria (≥ 300 mg of protein in 24-hour urine or in labstix isolated samples with two crosses in a single episode or two identifications of a cross). Preeclampsia was classified as severe when the hypertension was ≥ 160 × 100 mm Hg, creatinine ≥ 1.2 mg/dl, oliguria (< 400 ml within 24 hours), or signs of impending eclampsia were observed (nausea, vomiting, turbidity or visual scotomas, occipital headache and epigastralgia). The diagnostic criteria used at the time were ratified by the International Society for The Study of Hypertension in Pregnancy (ISSHP) in 2014.^16^

For the statistical analysis, the International Business Machines Statistical Package for the Social Sciences (IBM SPSS Statistics for Windows, IBM Corp., Armonk, NY, US) software, version 22.0, was used. The results were considered statistically significant when *p* < 0.05. The Pearson chi-squared and the Fisher exact tests were used for the categorical variables, and the Mann–Whitney U test was used for the continuous variables.

The project was approved by the Ethics in Research Committee of UFC under number 1.783.162. The free and informed consent forms of the women were not obtained individually. The information of interest was gathered from medical records without identifying the patient's name; instead, the record number was used.

## Results

The sociodemographic data of the cases classified as severe maternal morbidity with or without hypertension are shown in Table 1. No difference between the groups was observed, except regarding race/ethnicity, with three patients self-declaring as white, and 925 self-declaring as non-white. Most women did not have partners. Additionally, the women were between 20 and 34 years old, and had more than 8 years of schooling.

**Table 1:** Epidemiological and clinical characteristics of the patients identified as cases of severe maternal morbidity with and without hypertension

Hypertension			
Variables	Yes n (%)	No n (%)	*p*-value
*Age (years)*			0.061*
10–19	178 (21.5)	13 (12.9)	
20–34	511 (61.8)	74 (73.3)	
35 or older	138 (16.7)	14 (13.9)	
*Schooling*			0.081*
> 8 years	450 (63)	31 (51.7)	
≤ 8 years	264 (37)	29 (48.3)	
*Marital status*			0.603*
With a partner	194 (27.4)	18 (30.5)	
Without a partner	515 (72.6)	41 (69.5)	
*Pregnancies*			0.005*
1	403 (48.7)	32 (31.7)	
2–3	294 (35.6)	47 (46.5)	
> 3	130 (15.7)	22 (21.8)	
*Births*			0.336*
1	195 (53.1)	32 (53.3)	
2–3	138 (37.6)	19 (31.7)	
> 3	34 (9.3)	9 (15)	
*Previous cesarean sections*			0.832*
1	133 (70.4)	15 (68.2)	
≥ 2	56 (29.6)	7 (31.8)	
*Abortions*			0.024*
None	639 (77.5)	68 (67.3)	
> 1	186 (22.5)	33 (32.7)	
*Number of live births*			0.023*
1	197 (55.3)	29 (50)	
2–3	119 (33.4)	15 (25.9)	
> 3	40 (11.2)	14 (24.1)	
*Years since the last birth*			0.436*
1	4 (6.7)	18 (5)	
2–3	7 (11.7)	65 (18.1)	
> 3	49 (81.7)	276 (76.9)	
*Prenatal care visits*			0.092*
< 6	236 (44.4)	32 (56.1)	
≥ 6	295 (55.6)	25 (43.9)	

Note: *Pearson chi-squared test.

In relation to the obstetrical characteristics, there was a higher number of primiparous women (*p* = 0.005) and subjects without previous abortions (*p* = 0.024) in the group of patients with hypertension (Table 1). Most parturitions with hypertensive complications occurred through cesarean section: 635. Among the severe maternal morbidity indicators, there were 110 intensive care unit (ICU) admissions, 80 blood transfusions (29 requiring at least 5 units of packed red blood cells), 42 hospitalizations for more than 7 days postpartum, 33 hysterectomies, and 17 intubations (unrelated to anesthesia), with no significant difference between the groups with and without hypertension. Among the cases with potentially life-threatening conditions (*n* = 142, some patients had more than 1 condition), the following were highlighted: premature detachment of the placenta (*n* = 44); placenta accreta/percreta (10); shock (24); respiratory failure (23); sepsis (23); postpartum hemorrhage (23); endometritis (6); acute pulmonary edema (2); ectopic pregnancy (9); and uterine rupture (1), with no significant difference between the groups with and without hypertension. Magnesium sulfate was used in 682 cases, all of which were in the hypertensive group. In total, 51 participants with maternal near miss criteria were identified, 36 of whom were women with hypertensive disorders. From this group, 5 died; therefore, they were obviously excluded from the final near miss group. We observed 867 cases of non-near miss maternal morbidity criteria. During this period, there were 4,617 live births (LBs) in the institution that was studied, with a maternal near miss ratio of 55.55/1,000 LBs, a non-near miss maternal morbidity ratio of 190.6/1,000 LBs, and a maternal morbidity ratio of 238.2/1,000 LBs. We observed that the number of women at risk for death was 51, and the ratio was 11.2/1,000 LBs. Table 2 shows the maternal outcomes of the women classified with severe maternal morbidity with and without hypertension.

**Table 2:** Maternal near miss and death among the patients identified with severe maternal morbidity associated or not with hypertensive syndromes

Hypertension			
Variables	Yes n (%)	No n (%)	*p*-value
*Near miss criteria*			< 0,0007*
Yes	36 (71)	15 (29)	
No	86 (91)	81 (9)	
*Maternal death*			< 0.001*
Yes	5 (0.6)	5 (4.9)	
No	822 (99.4)	96 (95.1)	

Note: *Pearson chi-squared test.

Among the 10 cases of maternal death, all involved hospitalization in the ICU, and 7 patients received blood products. Among the cases with hypertensive syndromes, there were two cases of severe preeclampsia, and three of eclampsia. None of the patients with HELLP syndrome died. Sepsis was diagnosed in two of these cases. In the group without hypertension, four women died of hemorrhagic syndromes (two with premature placental detachment and two with a previous placenta accreta) and one case of severe sepsis was observed. Perinatal death was more prevalent in the group without hypertension (19.8% versus 5%, *p* < 0.001) (Table 3).

**Table 3:** Perinatal outcomes of women identified with severe maternal morbidity criteria with and without hypertensive syndromes

Hypertension			
Variables	Yes n (%)	No n (%)	*p*-value
*Perinatal death*			< 0.001*
Yes	41 (5)	20 (19.8)	
No	786 (95)	81 (80.2)	
*Average birth weight (g) (mean ± SD)*	2,723.6 ± 837.7	2,848.1 ± 742.3	0.569**
*Low birth weight*			0.817*
Yes	278 (36.8)	26 (38.2)	
No	477 (63.2)	42 (61.8)	
*Prematurity*			0.759*
< 37 weeks	281 (37.2)	27 (39.7)	
≥ 37 weeks	474 (62.8	41 (60.3)	

Notes: *Pearson chi-squared test; **Mann–Whitney test.

No significant differences in the near miss criteria between the participants with and without hypertensive disorders were observed, except for cyanosis (72% versus 28%, *p* = 0.038) and cardiopulmonary resuscitation (76% versus 24%, *p* = 0.011), which were more prevalent in the group with hypertension (Table 4).

**Table 4:** Maternal near miss criteria among the participants with and without hypertensive disorders

	Hypertension		
Clinical criteria	Yes n (%)	No n (%)	*p*-value
Acute cyanosis	20 (72)	8 (28)	0.038*
Respiratory rate > 40 or < 60 bpm	16 (70)	7 (30)	0.542*
Oliguria unresponsive to fluids and diuretics	19 (66)	8 (34)	0.197*
Shock	18 (75)	6 (25)	0.053*
Coagulation disorders	20 (71)	8 (29)	0.038*
Loss of consciousness for 12 hours or more	13 (62)	8 (38)	0.398*
Absence of consciousness and absence of pulsations or cardiac beats	15 (62)	9 (38)	0.333*
Cerebrovascular accident	17 (65)	9 (35)	0.704*
Uncontrolled convulsion	16 (62)	10 (38)	0.129*
Jaundice in the presence of preeclampsia	18 (64)	10 (36)	0.301*
Laboratorial criteria			
Saturation and oxygen < 90% for 60 minutes or more	18 (67)	9 (33)	0.516*
PaO_2_/FiO_2_ < 200 mm Hg	18 (67)	9 (33)	0.516*
Creatine ≥ 3.5 mm Hg/dL or ≥ 300 µMol/1	18 (67)	9 (33)	0.516*
Bilirubin ≥ 6.0 mg/dL or > 100 µMol/1	15 (60)	10 (40)	0.283*
pH < 7,1	20 (65)	11 (35)	0.112*
Acute thrombopenia (< 50,000/mm^3^)	11 (61)	7 (38)	0.641*
Handling criteria			
Continuous use of vasoactive drug	12 (75)	4 (25)	0.515*
Puerperal hysterectomy by infection and hemorrhage	23 (70)	10 (30)	0.882*
Transfusion ≥ 5 units of red-blood cell concentrate	22 (76)	7 (24)	0.127*
Intubation and ventilation for ≥ 60 minutes not related to anesthesia	13 (76)	4 (24)	0.389*
Dialysis for acute renal insufficiency	26 (76)	8 (24)	0.011*
Cardiopulmonary resuscitation (CPR)	21 (70)	9 (30)	0.849*

Note: *Pearson chi-squared test.

## Discussion

The present study evaluated severe maternal morbidity/maternal near miss in women with or without hypertension during pregnancy. It was performed in a reference center for obstetric and neonatal care over one year. We observed the presence of hypertension in most the participants (89.1%) who met the severe criteria.

Hypertensive syndromes are widely understood as the main cause of complications in prenatal care, occurring in 2% to 26% of gestations.^16^ ^17^ Hypertensive disorders are responsible for nearly 45,000 cases of maternal death every year worldwide, and are the main cause of maternal health in Latina America and the Caribbean. Estimates indicate that this problem represents a quarter of the total number of these deaths.^7^ ^8^ These observations make the study of the incidence of hypertension and its related causes relevant to understanding its relationship with maternal near miss.

Vidal et al^18^ performed a study whose goal was to identify the factors associated with maternal near miss in the city of Barbacena, state of Minas Gerais, Brazil. This study identified that in a sample of 276 women, which included 92 cases and 184 controls, the parturient women with a previous history of hypertension had a greater risk of developing severe maternal morbidity, such as preeclampsia and HELLP syndrome. In this group, 10.1% had severe hypertension, followed by 9.4% with severe preeclampsia, 0.4% with severe preeclampsia and severe hypertension, and 0.4% with eclampsia.

Studies point to hypertensive syndromes as frequent causes related to maternal near miss. In a study by Souza et al,^19^ 57% of the women had hypertensive disorders. In a study by Morse et al,^20^, nearly 70% of the women had preeclampsia, HELPP syndrome and eclampsia. This evidence emphasizes the need for and importance of prevention, early detection and treatment of these complications to control and reduce the risk of maternal near miss related to hypertensive causes.

We found that a lower frequency of the subjects in this population group evolved to more severe cases, as a lower frequency of subjects who met the maternal near miss criteria and a lower frequency of maternal and perinatal deaths were observed when compared with cases who met the morbidity criteria that were not associated with hypertensive disorders.

A possible explanation for these findings is that, as the two groups (with and without hypertension) were epidemiologically similar, there may be differences in the expertise of the institution's clinical body in managing cases with gestational hypertensive syndromes. The assistance protocols, which are often updated and based on the available scientific evidence, are widely disseminated in written form in virtual environments and on banners in every maternity sector where patients with hypertensive complications are cared for.

There was a prioritization in recent years of the dissemination of knowledge on gestational hypertensive pathologies with the development of multiprofessional protocols adapted to local realities, because this is the most prevalent pathology in cases of perinatal and maternal deaths. The magnesium sulfate protocols were associated with improvements in maternal indicators, as they are widely known and often implemented when they are recommended, and they lead to lower lethality in these cases. It is important to note that MEAC-UFC is the main reference point for gestational complications in the state of Ceará, Brazil – with a profile of following risky pregnancies.

The results of the present study showed that the maternal near miss ratio was of 55.55/1,000 LBs. These data are compatible with those found in the literature, which range from 0.7 to 101.7 cases per 1,000 births.^4^ ^5^ ^15^ Souza et al^19^ performed a study with 2,929 women, and identified a severe maternal morbidity ratio from 15 to 42 cases/1,000 births. In the same study, the estimated near miss morbidity was of 44.3/1,000 LBs. It is important to point out that most studies work with the number of births in the denominator of the maternal morbidity ratio, whereas the WHO recommends using LBs, as we have done in our study. The mortality ratio in the maternal near miss cases was of 18%, which is compatible with what is found in the literature^11^ ^19^ ^20^ ^21^ ^22^ for countries with low and medium socioeconomic levels.

It is necessary to develop a public policy to protect maternal and child health throughout all of the care levels to prevent complications or evolution into severe cases.^23^ A severe problem associated with the development of complications and the evolution of severity in the puerperal pregnancy cycle are delays in obstetrical assistance, whether they are related to patients, families, access difficulties, inefficient reference-counter reference systems, or care units and their staff (late diagnosis, delay in the appropriate and timely establishment of a therapy). An explanatory model was described by Thaddeus and Maine in 1994.^24^

Unfortunately, these delays were not evaluated in the present study, and they may explain the worse evolution and higher lethality found in the cases associated with hypertension when compared with the cases that did not show this association. Another cause of maternal morbidity/mortality are hemorrhagic syndromes, in which little time is available to avoid more severe complications and maternal and perinatal deaths.

A high frequency of cesarean sections (nearly 81%) was observed among our study participants. This result is similar to what was observed in other studies.^20^ It is assumed that this occurs due to the risk of complication in the clinical state of the patients.

In relation to prenatal care, most patients in the present study had fewer than six prenatal care visits. In a study by Mantel et al,^25^ the authors identified that between 21% and 30% of the women had not even been examined once during their prenatal care. When considering the results, it is important to note the number of visits that these pregnant women had. According to the WHO, the appropriate number of prenatal visits should be equal to or greater than six; however, this number may be lower in low-risk patients. Nevertheless, adequate prenatal assistance enables the early detection of and intervention for gestational complications, and it is a fundamental tool in reducing the risk of stillbirths and problems during pregnancy. The lack or the inadequacy of prenatal care is contemplated in level 1 and level 2 delays by Thaddeus and Maine.^24^

Some limitations in the present study must be recognized. The study was performed in a single unit; however, the maternity school is a reference for third-level care, and the sampling involved a significant number of patients. Another limitation was that the data were collected from records without applying a direct contact tool with the women, which could have broadened the range of information, such as information related to prenatal care and delays in the path through the health system when a patient needs hospital and medical care. We suggest that this tool be used in further studies.

The main limitation of the study is that the data collection period took place ∼ 9 years ago; however, data on near miss and maternal morbidity in the institution are now presented annually as a management report, and are very similar to those data that were collected. Additionally, these data were collected with a rigorous methodology, and were approved by the ethics committee for collection and therefore publication. These were factors for us in reporting this longstanding data.

The data found here have the potential to aid in the surveillance of maternal near miss cases and risk factors. They point out the importance of adequate prenatal care and assistance, as they present a determining factor in the reduction of the risk of hypertensive complications and subsequent cases of maternal near miss, which could be avoided.

## Conclusion

In summary, in the severe morbidity/maternal near miss population, the presence of hypertensive complications was very prevalent, constituting a risk factor for pregnant women and their fetuses. Among the women with hypertensive syndromes, we observed a lower presence of maternal near miss criteria and lower maternal and perinatal mortality rates when compared with women without hypertensive problems.
